# Cognitive Behavioral Therapy and Acceptance and Commitment Therapy for the Discontinuation of Long-Term Benzodiazepine Use in Insomnia and Anxiety Disorders

**DOI:** 10.3390/ijerph181910222

**Published:** 2021-09-28

**Authors:** Mélinée Chapoutot, Laure Peter-Derex, Hélène Bastuji, Wendy Leslie, Benjamin Schoendorff, Raphael Heinzer, Francesca Siclari, Alain Nicolas, Patrick Lemoine, Susan Higgins, Alexia Bourgeois, Guillaume T. Vallet, Royce Anders, Marc Ounnoughene, Jessica Spencer, Francesca Meloni, Benjamin Putois

**Affiliations:** 1 Association Nationale de Promotion des Connaissances sur Le Sommeil, 69001 Lyon, France; melinee@chapoutot.ch (M.C.); laure.peter-derex@chu-lyon.fr (L.P.-D.); patrick.lemoine99@free.fr (P.L.); 2 Lyon Neuroscience Research Centre, CNRS UMR 5292-INSERM U1028, Lyon 1 University, 69005 Bron, France; helene.bastuji@chu-lyon.fr (H.B.); dr.nicolas.sommeil@gmail.com (A.N.); 3 Sleep Medicine and Respiratory Disease Centre, Croix-Rousse Hospital, CHU of Lyon, 69004 Lyon, France; 4 Faculty of Medicine Lyon Sud-Charles Mérieux, Lyon 1 University, 69622 Villeurbanne, France; 5 Xceltranslate, 06000 Nice, France; wendyleslie06@gmail.com; 6 Institut de Psychologie Contextuelle, Montreal, QC H2Y 2S9, Canada; benji@contextpsy.com; 7 Centre D’Investigation et de Recherche Sur le Sommeil, CHUV, 1011 Lausanne, Switzerland; Raphael.Heinzer@chuv.ch (R.H.); Francesca.Siclari@chuv.ch (F.S.); 8 Centre Hospitalier Annecy Genevois, Service Pneumologie, 74000 Annecy, France; shiggins@ch-annecygenevois.fr; 9 Laboratory of Cognitive Neurorehabilitation, Faculty of Medicine, University of Geneva, 1201 Geneva, Switzerland; alexia.bourgeois@unidistance.ch; 10 LAPSCO, Université Clermont Auvergne, CNRS, LAPSCO, 63000 Clermont-Ferrand, France; guillaume.vallet@uca.fr; 11 Laboratoire EMC, Lyon 2 Université, 69500 Bron, France; royce.anders@univ-lyon2.fr; 12 Cabinet de Psychiatrie du Plateau de Haye, 54320 Maxéville, France; actiset@gmail.com; 13 Faculty of Psychology, Swiss Distance Learning University, 3900 Brig, Switzerland; jessica.spencer@etu.unidistance.ch (J.S.); francesca.meloni@etu.unidistance.ch (F.M.)

**Keywords:** benzodiazepine, drug withdrawal, benzodiazepine taper, cognitive behavioral therapy, acceptance and commitment therapy

## Abstract

Benzodiazepines have proven to be highly effective for treating insomnia and anxiety. Although considered safe when taken for a short period of time, a major risk–benefit dilemma arises in the context of long-term use, relating to addiction, withdrawal symptoms, and potential side effects. For these reasons, benzodiazepines are not recommended for treating chronic sleep disorders, anxiety disorders, nor for people over the age of 65, and withdrawal among long-term users is a public health issue. Indeed, only 5% of patients manage to discontinue using these drugs on their own. Even with the help of a general practitioner, this rate does not exceed 25 to 30% of patients, of which approximately 7% manage to remain drug-free in the long term. Cognitive Behavioral Therapies (CBT) offer a crucial solution to this problem, having been shown to increase abstinence success to 70–80%. This article examines traditional and novel CBT techniques in this regard, such as Acceptance and Commitment Therapy, which address both the underlying condition (insomnia/anxiety) and the substance-related disorder. The theoretical framework and evidence supporting the use of these approaches are reviewed. Finally, current research gaps are discussed, and key research perspectives are proposed.

## 1. Introduction

Benzodiazepines and related drugs (Z-drugs) (BZD) are a class of psychotropic drugs with an effect similar to that of alcohol: they amplify the activity of GABA-A receptors, producing hypnotic, anxiolytic, muscle relaxant, anticonvulsant, and amnesic effects [1]. BZDs are effective in the treatment of anxiety disorders [2,3,4] and insomnia [5,6]. Their duration of action depends on their elimination half-life (the time taken by the body to eliminate half of the dose), which is classified as short (1–12 h), medium (12–40 h), and long (>40 h). These substances require a prescription and are intended to be used under the surveillance of a medical practitioner. They are recommended by health authorities as a short-term treatment [7] and are therefore not intented to be used for the treatment of chronic anxiety or insomnia. However, such prescription recommendations, alone, have not prevented their widespread overuse, and the subsequent development of psychological addictions. This article aims to (i) describe the global epidemiology of BZD use with a particular focus on the situation in France, and the issues associated with long-term BZD use such as side effects, withdrawal syndrome and ecological impact, (ii) review the public health strategies proposed in European countries to limit the use of BZD as well as the therapeutic interventions available for promoting BZD withdrawal, and (iii) provide an up-to-date overview of the theoretical framework and the clinical trial-based evidence supporting the use of traditional and new generations of Cognitive and Behavioral Therapy (CBT) for successful long-term BZD use cessation, with a specific interest in Acceptance and Commitment Therapy (ACT).

## 2. Benzodiazepine Use and Abuse

### 2.1. Prevalence of BZD Use

#### 2.1.1. Statistics on BZD Consumption

BZDs are central nervous system depressants: they slow down brain activity, producing a relaxing or sedative effect. They are fast-acting and well-tolerated substances [4] which provide immediate relief. They have been shown to be effective in the acute treatment of generalized anxiety disorder, social anxiety, panic disorder, and insomnia [8]. The French High Authority for Health (HAS) recommends a maximum period of use of 4 weeks in the treatment of insomnia, and 12 weeks for anxiety. However, BZDs continue to be used when these disorders persist, leading to the problem of dependence and abuse of these substances in the context of chronic anxiety and insomnia, which is the subject of this article.

France and Spain are the countries in Europe with the highest consumption of BZDs. In 2015, 10.3 % of the French population had taken a benzodiazepine at least once for anxiety and 5.6% for hypnotic purposes [9]. Misuse of BZDs refers to their use without a prescription, in higher doses or frequency than prescribed, or in reference to recreational use. BZD misuse is reported in approximately 2% of the general adult population [10,11]. Since addiction occurs within the first month of use [12], the legal prescription period is often exceeded [9,13,14]. In France, 13.5% of consumers use these drugs beyond the legal time limit [9]. Moreover, one in three people over age 65 consume them regularly [15]. A study of BZD use among 520,000 swiss patients shows that 14.5% of patients have been using BZD beyond 12 months, and like in France, this overuse tends to increase with age [16]. However, these prevalences are low compared to tobacco or alcohol addictions, which suggests that medical prescription of these psychoactive substances is a limiting factor, although it does not completely protect individuals against misuse.

#### 2.1.2. Consumption Trends

Despite recommendations, the number of prescriptions has increased exponentially over the last 30 years [17]. In the United States for example, the number of people receiving at least one prescription of BZD per year increased by 67% between 1990 and 2013, and the doses prescribed more than tripled during this period. Deaths from BZD overdoses have increased by more than 400%, and visits to emergency facilities for BZD prescriptions increased by more than 300% between 2004 and 2011 [18]. The proportion of BZD-using drivers involved in accidents has also increased over the past 10 years [19].

France saw a slight decrease in consumption (5.7%) between 2012 and 2015 following HAS campaigns to reduce prescriptions [9], but the prevalence remains nevertheless very high (13.4%). During the COVID-19 pandemic induced lockdown period of 2020, initiation of new BZD treatments in France increased by 8% compared to 2019, “despite the relative difficulty of consulting a doctor, or even the fear of disturbing them or the risk of contagion beyond teleconsultations” [20].

### 2.2. Low Benefit-to-Risk Ratio

#### 2.2.1. Side Effects

BZDs are contraindicated in cases of sleep apnea syndrome, respiratory disorders, history of drug or alcohol abuse, severe personality disorders, or psychotic illness [21]. Given the high prevalence of sleep apnea as a comorbidity in insomnia disorders [22], systematically responding to sleep complaints with benzodiazepine prescriptions should be avoided.

The prescription and use of BZDs has been under scrutiny for over 40 years [23] due to their many side effects [8,24,25]. In the short term, they increase daytime sleepiness and the risk of road accidents [26,27]. They can cause muscle weakness, loss of coordination and gait problems, dizziness, confusion, speech problems, and even anterograde amnesia [15]. Seizures [28,29,30] have been reported in cases of abuse and withdrawal. BZD use should be avoided by pregnant and breastfeeding women [31]. In the elderly, long-term use of BZDs increases the risk of falls and hip fractures, road accidents, cognitive decline, and mortality [32,33,34]. However, the link between BZD and the development of neurodegenerative diseases is still questioned [34,35]. BZDs are associated with an increased risk of mortality when combined with opioids [36] and should not be consumed with alcohol.

Tolerance to BZDs develops rapidly, followed by physical and psychological dependence, often creating conditions leading to doses being increased and their chronic use or abuse. Long-term use may result in altered sleep structure (reduced slow wave sleep), as well as cognitive dysfunction (especially with high doses: reduced attentional resources and impaired verbal and visuospatial memory, working memory, and executive functions) [25].

#### 2.2.2. Withdrawal Syndrome

Spontaneous attempts to discontinue consumption cause withdrawal syndrome in 50–80% of users [37,38], characterized by increased insomnia or anxiety (i.e., rebound anxiety/insomnia), irritability, headaches, muscle and stomach pains, sensory hypersensitivity, major weight loss, and seizures. These symptoms are more severe following withdrawal from short rather than long half-life BZDs [39,40,41,42,43]. Withdrawal syndrome is chemical in nature, but is strongly influenced by psychological factors [38] and its manifestation depends on the user’s personality [44]. Benzodiazepine withdrawal syndrome may develop at any time, usually around 4–7 days but up to 3 weeks after stopping long-acting BZDs [45], and within 48 h after reducing intake of short-acting BZDs [46]. These symptoms often lead the user to relapse, reinforcing the psychological dependence.

#### 2.2.3. Ecological Impact

The human body does not metabolise BZDs and releases them through the urine. Excessive concentrations of these drugs in waterways are considered to be a threat to aquatic ecosystems [47]. A study published in the Science journal reveals that concentrations of benzodiazepines in water effluents affect the behavior of fish and disrupt the food chain, with potentially serious evolutionary consequences [48].

### 2.3. Recommendations for Use

#### 2.3.1. Reduce Access to BZDs

Reducing access to these medications is one strategy to limit addiction and substance abuse. In the case of BZDs, only medical prescriptions can (in theory) provide access. Educating doctors and patients about reasonable consumption is therefore fundamental. In recent years, French and British health authorities [7,49,50] have recommended prescribing these products at minimal doses and for the shortest possible duration, set at less than 12 weeks for anxiety disorders and 4 weeks for insomnia. These timeframes include the period of dosage reduction recommended to prevent the onset of a withdrawal syndrome. In Switzerland, BZDs are listed as controlled substances under the Federal Law on Narcotics and Psychotropic Substances (Law on Narcotics, LStup 2018), in the same way as narcotics.

#### 2.3.2. Increase the Cost of BZDs

Increasing the cost of these medications is another strategy to reduce their rate of abuse, as was done with tobacco [51]. In France, since 2014, seven BZDs were practically withdrawn from insurance reimbursement programs, in which patient copay rates significantly increased from 15% to 85%. The objective of this measure was to “reduce long-term prescriptions of BZDs and related drugs for anxiety and insomnia, due to an adverse benefit–risk ratio”. However, given that the price of 30 tablets is only 3 to 5 euros, the monthly budget for BZD consumers remains minimal despite this effort.

#### 2.3.3. Deprescribing

Withdrawal from BZDs is especially recommended for long-term users over the age of 65. In France [52,53] and in Canada [54,55], awareness campaigns have been carried out and withdrawal guides distributed. Their effectiveness is difficult to estimate.

Consumption must not be reduced abruptly. Studies assessing the effect of abrupt versus gradual withdrawal [43,53,54,55,56] show that the withdrawal syndrome observed in the case of abrupt discontinuation makes it very difficult to stop the use of these molecules, especially in the case of short half-life BZDs. These studies recommend reducing BZD consumption over a period of at least 4 weeks. Some studies have reported epileptic seizures [57] following an abrupt discontinuation of BZDs, and hence recommend a gradual reduction over at least 6 days to avoid convulsions [58].

The use of substitute substances with a longer half-life such as diazepam, or less addictive substances such as melatonin [59,60,61,62,63,64,65] is often proposed in routine treatment. Nevertheless, two reviews show no added value of melatonin substitution [66,67] and, to the best of our knowledge, arguments regarding the benefit of diazepam substitution are mostly theoretical (not evidence-based).

Many brief intervention strategies have been tested for BZD discontinuation. These can take the form of a discussion with the prescribing doctor [68,69,70,71,72], a simple letter from the doctor recommending that the treatment should be stopped or gradually reduced [70,71,73,74,75,76], therapeutic education sessions with a nurse [65], or an educational brochure about withdrawal distributed by the pharmacist [77,78]. Randomised controlled trials (RCT) have shown an overall success rate of 25–40% for this type of intervention, depending on the study, versus 5% for waiting list (WL) or treatment as usual (TAU). Several publications [24,79,80,81,82,83] argue that these brief interventions are very effective, doubling or tripling the abstinence rate achieved in standard care. However, this absolute rate remains low overall, with abstinence being achieved in less than half of patients. Moreover, studies of brief interventions only report relatively short follow-ups (generally between 6 and 12 months), while the relapse rate is potentially high. For example, one study reported an abstinence rate of 24% at 6 months following a simple letter from the prescribing doctor [73], and this rate fell to 7% ten years later [74]. Based on these meta-analyses, withdrawal from BZD may therefore wrongly appear to be easy. The last two meta-analyses on the topic [84,85] emphasise the effectiveness of supervised withdrawal supported by psychotherapy and in particular the addition of cognitive behavioral therapies.

## 3. Cognitive Behavioral Therapy (CBT) for Discontinuing Long-Term Benzodiazepine Use in Insomnia and Anxiety Disorders

CBT for the treatment of BZD addiction is divided into three stages: (1) regulating the causes of BZD use by treating insomnia and anxiety, (2) applying the knowledge gained in the first stage to boost compliance with the taper program and management of withdrawal syndromes, (3) preventing relapse by adopting regulatory behaviors and cognitive strategies rather than systematic use of psychoactive substances.

### 3.1. Methods

Medline, PsycINFO, and the Cochrane Collaboration Central Register of Controlled Trials were searched in November 2020 using the following terms: [anxiety OR alprazolam OR anxiolytic * OR benzodiaz * OR bromazepam OR clobazam OR clonazepam OR diazepam OR flunitrazepam OR hypnotic * OR insomnia OR lorazepam OR midazolam OR nitrazepam OR oxazepam OR temazepam OR triazolam OR zolpidem OR zopiclone] AND [abuse OR addiction OR cbt OR cessation OR chronic OR dependen * OR discontinue * OR long-term OR misuse OR overuse OR reduc * OR taper * OR withdraw *] AND [therap * OR cbt OR act OR cognitive behavioral therapy OR acceptance commitment therapy]. Studies were also identified from citations in studies, reviews, and meta-analyses of interventions which aimed to reduce benzodiazepine use.

### 3.2. Effectiveness of CBT on the Causes of BZD Use

The main advantage of using CBT in the treatment of BZD addiction is that it targets not only the addiction itself, but also the underlying conditions that resulted in the use of the BZD, namely insomnia and anxiety. By treating the causes which led to the prescription of BZD, consumers increase their chances of being able to discontinue use. However, studies on the effectiveness of brief interventions do not take into account the link between BZD use and the initial pathology, and indeed rarely include a diagnosis of these or other specific pathologies. Addiction to BZD may certainly be a disorder in itself (i.e., primary disorder), aggravated by the duration of use and the half-life of the drug, which are among the predictors of withdrawal failure; but it is the psychopathological variables [86,87,88,89,90,91] (e.g., psychological suffering, anxiety or insomnia) which best predict relapse rates in withdrawal programs.

By treating the mental pathologies that lead to BZD use, CBT can increase the chances of successful withdrawal, as it provides the patient with alternative coping strategies to BZD consumption.

CBT for insomnia (CBT-I) includes a range of methods aimed to improve sleep and manage dysfunctional thoughts related to the anxiety of not sleeping [92] such as sleep restriction, stimulus control, paradoxical intention, relaxation, and cognitive restructuring. CBT-I is recognised as being the treatment of choice for insomnia [93], both in isolated form or as a comorbidity [94], in psychiatric populations [95], among the elderly [96,97], and delivered remotely [98]. Three RCTs [99,100,101] which compared CBT-I, BZD and placebo for chronic insomnia all show that CBT-I was the most effective sleep intervention. Notably, pharmacotherapy produced only moderate improvements that were limited to the duration of drug administration. Moreover, CBT-I proved to be cost-effective compared to pharmacotherapy or no treatment [102].

Regarding the treatment of anxiety disorders, meta-analyses show overall good efficacy of CBT [103,104] in the treatment of health anxiety (large effect size vs. various control conditions including waiting list, medication and psychological therapies, d = 1.01) [105], panic disorder (large effect vs. no treatment (d = 0.83), small effect vs. other psychotherapies (d = 0.05)) [106], and generalized anxiety disorder (large effect vs. control condition, mostly waiting list, d = 0.84) [103]. CBT also works well when delivered remotely [107], and to a lesser extent in the elderly [108,109]. The components of CBT in the treatment of panic disorders are [110,111]: psychoeducation, a somatic skills component (e.g., breathing retraining), relaxation, cognitive restructuring, and interoceptive exposure. For generalized anxiety disorder [112], the techniques include cognitive restructuring, problem-solving training, cognitive exposure to worries, situational exposure, and relapse prevention.

### 3.3. Benefits of CBT for Optimising Adherence and Compliance with the Withdrawal Program

Successful withdrawal also depends on the individual’s level of confidence in their ability to manage their psychological states without the use of substances [113,114,115]. By reducing the severity of anxiety or insomnia, CBT can reinforce the patient’s sense of self-efficacy, enabling them to increase their confidence in their ability to withdraw from BZD. In addition, the development of emotional regulation abilities and cognitive and behavioral work enables them to better manage their withdrawal symptoms, the intensity of which also increases the risk of relapse [86].

Furthermore, CBT provides a concrete and pragmatic framework for the development of effective strategies to achieve objectives. By encouraging the patient to take an active role, this goal-oriented approach increases compliance with the withdrawal program and reduces the risk of early relapse [38,44,86,87,91,116].

For the treatment of addictions, CBT is based on the transtheoretical model of behavioral change [117], which proposes that health behavior change involves progress through six stages of change: precontemplation (no action is intended to be taken), contemplation (a change is planned in the next 6 months), preparation (a change is planned in the immediate future), action (changes have been made in the past 6 months), maintenance (work is done to prevent relapse) and termination (no temptation anymore and 100% self-efficacy). Health promotion programs based on stage-matched interventions have proved to be effective in several fields [117]. This model is particularly well suited to the issue of BZD addiction. The stage of precontemplation in BZD addiction is distinct from that of other substances, as BZDs are prescribed by a physician. The patients see the substance as a drug that helps them and not as an addictive substance on which they are dependent. At this stage, awareness campaigns and psycho-education by doctors or pharmacists are useful. In the contemplation stage, prior unsuccessful attempts to stop using BZD, as well as the memory of rebound symptoms of the anxiety or insomnia, reinforce the patient’s feeling of inefficacy. In the context of secondary substance-related disorders (SRD), CBT targeting anxiety and insomnia is necessary at this stage before moving on to the preparation stage. In the action stage, all learned CBT methods are applied to control withdrawal symptoms (sleep restriction, stimulus control, relaxation, cognitive restructuration, panic attack control, in vivo exposure). The cognitive restructuring tools allow patients to observe the fact that there is no linear correlation between the reduction of BZD consumption and the occurrence of withdrawal symptoms. They also help them to differentiate between the drug’s actual effects and its placebo effects. When patients become drug-free, CBT advises booster sessions in order to maintain behavioral and cognitive skills and prevent relapse.

### 3.4. Effectiveness of Adding CBT to Taper Programs

To date, eight RCTs (see Table 1) comparing the effectiveness of a taper program alone versus a taper program associated with CBT [33,112,118,119,120,121,122,123] and two publications reporting their long-term outcomes (>24 months) have been published [124,125]. RCT designs and results are summarized in the table.

Dose reduction programs usually include information about withdrawal and the effects of discontinuing treatment, a dose reduction schedule that sets out rules for reduction and is actively developed by the patient, and weekly interviews to monitor withdrawal symptoms and provide support throughout withdrawal. These brief interviews (10–15 min) are carried out by a physician or healthcare staff. The parameters of the withdrawal schedule vary from study to study: The reduction rate generally ranges between 12.5–25% of the dosage every 2–21 days for a total reduction period ranging between 4–21 weeks (usually 8 weeks). Only two studies propose a stepwise increase in drug-free days/nights [33,123]. In most studies, withdrawal schedules are individualized according to the half-life of the benzodiazepine, dose, timetable, and motivation of the patient. Morin et al. [33] very precisely described the principles in designing the schedules with the patient’s collaboration: “(1) setting goals, (2) stabilization with the use of a single benzodiazepine for patients who are using more than one, (3) reduction of about 25% of the initial dosage every 2 weeks until the lowest available dose of the benzodiazepine is reached, (4) introduction of an increasing number of drug-free nights, and (5) schedule hypnotic use rather than use on an as-needed basis. The specific dose reductions vary as a function of patients’ readiness to discontinue medication and the presence or absence of withdrawal symptoms. However, the time-limited nature of this program is emphasized by setting anchor points”.

The CBT used in these protocols, with the exception of one study [121], principally targeted the conditions that initially led to the misuse of BZD: insomnia in people over 50 years old [33,122,123], panic disorders [118,119,120], or generalized anxiety disorder (GAD) [108,124]. Generally, patients attended between 8 and 12 weekly sessions lasting 60–90 min of psychotherapy in a group or individual setting, and patients were not encouraged to start reducing their BZD consumption until the 3rd or 4th session.

Five out of seven studies have demonstrated greater success of taper programs in combination with CBT than taper programs alone: for panic disorders, CBT doubles (80% versus 40%) [120], triples (75% versus 23%) [118], or quadruples (62.5% versus 12.5%) [119] the number of drug-free patients at 6 months. For GAD, CBT doubles (75% versus 35%) the rate of abstinence at 12 months. Among insomniacs, CBT doubles (85% versus 48%) [33] or triples (70% versus 25%) [122] the number of drug-free patients at 12 months. Among patients suffering from panic disorder, the rate of abstinence is maintained at 24 months (75% versus 30%) [125]. For insomnia, relapses are observed at 5 years, but the greater success of CBT is maintained (50% versus 32%) [124].

Two studies showed no added value of CBT for withdrawal. The first (50% versus 43%) provided CBT-I without the main therapeutic component of sleep restriction [123]. The second study (62% versus 58%) [121] targeted neither insomnia nor anxiety but “supported the participants during the tapering-off process and in preventing relapse” and focused on withdrawal symptoms only. The lack of efficacy of CBT in these studies could be explained by the fact that, as the intervention targeted only the SRD and not the underlying pathology, discontinuing consumption of the substance left patients without a coping strategy to manage the symptoms associated with the initial or root condition. This is difficult to assess, however, as follow-up stopped at three months post-treatment and CBT intervention “started halfway through the tapering-off period”, which seems inadequate in view of the transtheoretical model of behavioral change [117].

The number of studies is too small to firmly conclude that CBT is more effective than withdrawal programs alone [126]. However, some meta-analyses show that CBT at least doubles the abstinence rate compared to a simple withdrawal program [82,127,128]. As this control condition is itself more effective than WL or TAU, which have a success rate of only 5%, we can suppose that the effectiveness of CBT compared to brief interventions (which include WL or TAU as a control condition) is clearly underestimated in the meta-analyses on the treatment of BZD use disorder.

## 4. Acceptance and Commitment Therapy for Discontinuing Long-Term Benzodiazepine Use

Acceptance and Commitment Therapy (ACT) is a third generation CBT, which promotes values-congruent behaviors (commitment) over reducing unpleasant private experiences or symptoms (control) [129,130]. Therapeutic work in ACT is structured around six processes: contact with the present moment (mindfulness), acceptance (making space for unpleasant feelings), defusion (distancing oneself from problematic thought patterns), values (what is important), committed action (acting in line with what is important), and context (adopting an observer perspective on one’s experience).

ACT promotes acceptance of inner experiences (thoughts, emotions, sensations) rather than avoidance or regulation of these experiences. Taking benzodiazepines often serves to avoid the experience of stress, anxiety, insomnia, and anxious anticipation of panic attacks or insomnia. The effects of such use are more acute (and therefore reinforcing) the shorter the half-life of the substance. In line with the Relational Frame Theory [131], the theoretical underpinning of ACT, some verbal contexts, such as taking one’s thoughts about insomnia or anxiety literally, can feed experiential avoidance, thus leading to BZD use. ACT processes such as acceptance and defusion can undermine the dominance of such contexts and reduce the likelihood of substance use. ACT may be effective in the treatment of addictions [132,133] by increasing psychological flexibility, i.e., the ability to choose value-guided behavior even in the presence of problematic thoughts and emotions. ACT aims to broaden an individual’s range of behavioral options when faced with symptoms, in this case by promoting alternative behaviors to BZD consumption.

### 4.1. Differences between CBT and ACT

As a third wave behavioral-cognitive therapy, ACT proposes to broaden the goal of therapy away from changing thoughts and regulating emotions and towards increasing value-consistent behavior. By doing so, it aims to reduce the functional importance of particularly tough patterns regarding insomnia/anxiety and cravings by placing them in a context in which they are seen as merely thoughts and physical sensations rather than causes of consumption. At the same time, ACT helps to orient oneself according to the motivating functions of values, behaving like the person one wants to be, thus increasing value-congruent behavior, and augmenting one’s sense of satisfaction with their life and a sense of broader choices. This appears to be a significant departure from traditional CBT conceptualizations, which view thoughts and feelings as causes and therefore aim to change and regulate them. Overall, ACT teaches CBT processes but in a more flexible context by promoting approach and expansion behavior (rather than avoidance and constraints) [134]. In CBT-I, sleep restriction and stimulus control are applied in order to improve sleep (which still focuses patients on their sleep problem). In ACT-I, these methods are presented to optimize time devoted to committed actions and to improving quality of life (reducing insomnia). In CBT-I, withdrawal is presented in response to the fear of side effects; in ACT-I, withdrawal is presented in concordance with health-related values such as a feeling of being free or being “myself”. In ACT-I, patients are encouraged to take their benzodiazepine mindfully (which would reduce the automatic process of consumption) with acceptance, in contrast to CBT-I where patients consume with aversion.

### 4.2. Effectiveness of ACT on the Causes of Benzodiazepine Use

ACT is a transdiagnostic approach [129] the benefit of which has been documented in disorders leading to BZD use, with proven efficacy in insomnia [135] and probable efficacy in anxiety disorders [136,137,138].

Insomnia is a notable symptom of withdrawal syndromes and a factor of relapse as emphasized by many studies [38,88,90]. ACT implements processes which promote sleep [139]: acceptance and mindfulness processes reduce insomniacs’ efforts to control their sleep and fight against fatigue and their attempts to sleep on command by taking sleeping pills. In this way, it facilitates the application of sleep restriction, a key instruction in CBT programs for insomnia. ACT focuses on the therapeutic goal of living better, not just sleeping better, which prevents insomniacs from obsessing about sleep and thus facilitates falling asleep. There have been only few RCTs specifically evaluating ACT for insomnia [134,140,141,142]. Nevertheless, many studies have demonstrated a positive effect of ACT on insomnia symptoms in various pathologies. A meta-analysis of 19 studies and 1577 participants [135] reported improved sleep and reduced insomnia following an ACT intervention for chronic fatigue [143,144], tinnitus, or chronic pain [145,146].

In ACT, patients learn to end the struggle with their anxiety-related discomfort and take charge by engaging in commitment actions in accordance with their values. ACT expands the context of interoceptive exposure for the purpose of symptom suppression, to include much more appealing goals: living life in harmony with our values. We can assume that ACT facilitates the long-term use of traditional CBT methods. Mindfulness reduces the paradoxical effect by suppressing anxiety-provoking thoughts and stress [147,148]. A 2013 meta-analysis of 38 studies and 323 participants suggested that ACT was effective in the treatment of anxiety, but noted that there were still too few RCTs on the subject [136], with the most compelling results being observed for mixed anxiety and social anxiety issues. More recently, another meta-analysis found that internet-delivered ACT for adults could be an efficacious and acceptable treatment for adults with GAD and general anxiety symptoms [138].

### 4.3. Effectiveness of ACT for Substance Use Disorders

Two reviews have confirmed the effectiveness of ACT in the treatment of substance use disorders (alcohol, tobacco, opioids, and metamphetamins) [132,133]. ACT has a significant effect on maintaining the abstinence rate. Only three studies have compared ACT with CBT and these have shown equivalent effectiveness in the short-term [149,150,151]. In the long-term, ACT was more successful than CBT in maintaining abstinence rates [150,151]. ACT increases acceptance of physical cravings [150] and would appear to be generalized to other psychopathological problems [151]. It should be noted, however, that the number of studies available is still insufficient to establish a recommendation.

### 4.4. Effectiveness of Adding ACT to Taper Programs

To the best of our knowledge, the effectiveness of ACT in reducing BZD use has only been assessed in one pilot study of insomnia associated with sleeping pill addiction [137]. No study has assessed the effectiveness of ACT on BZD withdrawal as a primary outcome.

### 4.5. The Benefits of ACT for Adherence and Compliance with Withdrawal Programs

To date, there have been no studies that have assessed the efficacy of ACT for treating BZD addiction. How might ACT be an appropriate intervention for this disorder? In accordance with the transtheoretical model of stages of change, by encouraging value-driven actions, ACT targets the patient’s ambivalence about using a chemical substance to regulate their inner states and facilitates the transition from the contemplation stage to the planning stage. Many BZD addicts would ideally like to not use any substance. At the action stage, increasing acceptance of inner experiences facilitates tolerance of withdrawal symptoms to promote continued compliance with withdrawal. Cognitive defusion and mindfulness skills help the patient maintain goal-directed behaviors, in this case withdrawal, and to resist taking the drug in the event of insomnia or rebound anxiety. During the phase of maintaining abstinence, by orientating behaviors towards life-values, ACT improves quality of life which, along with social support, is a factor involved in successful withdrawal and protection against relapse [113,115,152,153,154]. Promoting psychological flexibility encourages flexible use of BZD, for example by restricting their consumption to specific, time-limited contexts, thereby reducing the risk of relapse into chronic use.

## 5. Discussion

BZD are legal psychoactive substances obtainable through a prescription and under medical supervision. However, these constraints do not prevent their overconsumption, abuse, and the development of subsequent addiction. The studies reviewed herein are clear: 95% of long-term users are unable to withdraw from benzodiazepine consumption. A brief medical or paramedical intervention can help, but the abstinence rate peaks at 40%. For long-term users, withdrawal from BZD use is very difficult. In this context, CBT offers a doubly interesting alternative: it addresses the main causes of use (anxiety disorder and insomnia) and teaches patients methods of emotional and cognitive regulation, problem-solving and goal-directed behaviors which help to initiate, complete, and maintain a long-term reduction or cessation of BZD use.

The study of treatment for BZD addiction therefore sheds light on the importance of not “putting the cart before the horse”: it is necessary to arm patients before they go to war against the addictive substance to prevent the risk of relapse. Can we generalize this reflection to other SRDs? Undoubtedly. There is thus a need to differentiate between primary and secondary SRDs. This differential diagnosis is rarely made in research that does not focus on specific pathologies, particularly those regarding brief interventions. In the case of BZD, it can be assumed that a taper program alone is sufficient for primary addictions and insufficient for addictions secondary to another mental disorder, for which it is necessary to combine withdrawal with a pathology-specific CBT.

To date, there is no manualized and standardized CBT program specifically targeting BZD use disorder alone, although a proposal has been made in this vein, but without benefits compared with short interventions [121]. From a CBT perspective, such a program should include elements of anxiety and sleep management. ACT represents a promising, transdiagnostic approach, targeting the experiential avoidance that is a central component of BZD addiction.

Studies on CBT interventions are scarce: nine RCTs over 30 years of research. The parameters of withdrawal programs also vary greatly from one study to another. Research consensus and methodological recommendations for research are lacking (a definition of the long-term user, a precise description of the withdrawal program, psychopathological diagnosis at the inclusion stage, taking into account the half-life of the substance, etc.). These would eventually make it possible to specify institutional recommendations on deprescribing, which currently remain too broad (e.g., the recommended period of withdrawal varies between 4 weeks and one year).

The number of studies on CBT treatment of insomnia and anxiety is much larger. We can only encourage such studies to assess BZD use not just as a secondary outcome, but as a primary outcome using all the necessary measures.

In order to make the brief interventions and CBT comparable, a common control condition should be added: i.e., CBT + BZD taper should be compared to BZD taper alone and to WL. By having a common control condition (WL), the effect sizes would then be comparable for any type of intervention for BZD reduction. In view of the very high relapse rate, only studies with a minimum follow-up of 12 months should be included in the scientific debate. Long-term follow-ups (>2 years) are too scarce.

The prescription of BZDs represents a quick and easy response for primary care physicians: in primary prevention, as they do not know where to refer their patients for a problem of anxiety or chronic insomnia, or in secondary prevention, for the problem of withdrawal from these substances. Psychotherapy also represents a certain investment of time and money for patients, even if it is cost-effective in the long term. Brief interventions are inexpensive and are successful for some patients. Other patients, on the other hand, will not manage to wean themselves off BZDs without psychotherapy. It would therefore be useful to be able to identify the psychological profiles of patients likely to benefit more from a particular intervention. This personalized care would enable the clinical pathway to be optimized and health costs to be rationalized. The question would then no longer be whether CBT/ACT are effective therapies, but rather for which patients they are necessary for successful withdrawal.

## 6. Conclusions

Withdrawal from BZDs among long-term users is very difficult. CBT, whether of the traditional or new generation approach, increases the chances of successful withdrawal from BZDs. Such therapies should be mandatory for patients with anxiety disorders or chronic insomnia before starting to reduce their medication. Sleeping better, feeling less anxious about going to bed or not sleeping, controlling stress, regulating worries, and managing panic attacks seem to be prerequisites for embarking on the discontinuation of a drug which helps to chemically palliate these problems.

## Figures and Tables

**Table 1:** Randomized Controlled Trials comparing the effectiveness of a taper program alone versus a taper program associated with CBT.

Study	Condition	Population	Benzodiazepine Use	Design	Withdrawal Schedule	Main Outcomes
Otto et al., 1993 [118]	Panic disorder	n = 33, 22 women	Alprazolam or clonazepam use >6 months	Slow taper alone (5 to 7 weeks) (n = 16) vs. slow taper + 10 weekly sessions (60–90 min) of group CBT (n = 17)	Alprazolam: Reduction of 0.25 mg or 0.125 mg every 2 days (depending on the initial dose) Clonazepam: reduction of 0.25 mg every 4 or 8 days (depending on the initial dose)	Successful discontinuation (=completion of the taper and no use of BZD beyond “minimal p.r.n use” during the 2 post-discontinuation weeks) in 76% with CBT vs. 25% without CBT (*p* < 0.01) At 3 months: persistent effect of CBT
Spiegel et al., 1994 [120]	Panic disorder	n = 21, 17 women	Alprazolam use, 1 to 10 mg/j	Supportive drug maintenance and slow flexible taper (n = 10) vs. same taper + 12 weekly sessions of individual CBT (n = 11)	Reduction of 0.125 mg to 0.5 mg/1–2 week, mean duration 6.5 weeks	Successful discontinuation (=completion of the taper and no use of BZD through the follow-up) 2 weeks after treatment: 90% with CBT vs. 80% without CBT (ns) At 6 months: 90% with CBT vs. 40% without CBT (*p* < 0.05)
Baillargeon et al., 2003 [122]	Chronic insomnia in older adults	n = 65, >50 years, 38 women	Daily use of BZD >3 months (Molecules not specified)	Slow taper alone (n = 30) vs. slow taper + 8 weekly sessions (90 min) of group CBT (n = 35)	25% reduction of dosage every 1–2 weeks	Successful discontinuation (= BZD cessation confirmed by blood screening) in 77% with CBT vs. 38% without CBT after treatment completion (*p* = 0.002), and results maintained at 3 and 12 months
Voshaar et al., 2003 [121]	Not mentioned	n = 180, 128 women	BZD use >3 months BZDs were switched for an equivalent dose of diazepam	Usual care (letter with advice to stop) (n = 34) vs. taper (n = 73) vs. taper + 5 weekly sessions (120 min) of group CBT (n = 73)	25% reduction of dosage every week	Successful discontinuation (=no self-reported BZD use at 3 months follow-up) in 58% with CBT vs. 62% tapering off without CBT (no additional benefice of CBT) vs. 21% with usual care
Morin et al., 2004 [33]	Chronic insomnia in older adults	n = 76, >55 years, 38 women	BZD use >50% of nights >3 months (different molecules: lorazepam, alprazolam, bromazepam, oxazepam, temazepam, clonazepam, flurazepam, triazolam)	Supervised withdrawal program (n = 25) vs. CBT for insomnia (weekly 90 min sessions) (n = 24) vs. supervised withdrawal program + CBT (n = 27) For all groups: program duration 10 weeks	25% reduction of dosage every 2 weeks and introduction of an increasing number of drug-free nights	Drug-free patients (confirmed by blood and urine samples): 85% taper + CBT vs. 48% taper alone vs. 54% CBT alone at post-treatment (*p* < 0.002) and results maintained at 3 and 12 months Reduction of weekly quantity of BZD use (dosage_overall 90% reduction_and number of nights_overall 80% reduction) in the 3 groups with lower frequency of medicated night in the CBT + taper vs. taper alone group
Gosselin et al., 2006 [112]	Generalized Anxiety Disorders (GAD)	n = 61, 36 women	BZD use >4 days/week for >12 months (different molecules: clonazepam, lorazepam, alprazolam, bromazepam, oxazepam, temazepam, diazepam, clorazepate)	Non-specific psychological treatment (NST) + taper (n = 30) vs. CBT + taper (n = 31), 12 weekly 90 min sessions	25% reduction of dosage every 2–3 weeks	Drug-free patients at post-treatment: 74% CBT + taper vs. 37% NST + taper group, *p* < 0.001. Results maintained at 3, 6 and 12 months. Greater proportion of patients no longer with GAD criteria in the CBT group
Otto et al., 2010 [119]	Panic Disorders	n = 47, 31 women	Alprazolam or clonazepam use >6 months	Taper alone (5 to 9 weeks) (n = 15) vs. taper + CBT (8 weekly 60–90 min sessions followed by 3 booster sessions separated by 2 weeks) (n = 16) vs. taper + relaxation (same number/duration of session as CBT sessions) (n = 16)	Alprazolam: Reduction of 0.25 mg or 0.125 mg every 2 days (depending on the initial dose) Clonazepam: reduction of 0.25 mg every 4 or 8 days (depending on the initial dose)	Successful discontinuation (=completion of the taper and no use of BZD beyond “minimal p.r.n use” during the month post-discontinuation) in 56% CBT vs. 31% relaxation vs. 40% taper alone (ns), and maintained at 3 (44% vs. 13% vs. 27%, ns) and 6 months (63% vs. 13% vs. 27%, *p* < 0.01)
Lichstein et al., 2013 [123]	Chronic insomnia in older adults	n = 70, >50 years, women	Hypnotic dependance (BZD, non-BZD receptor agonists, sedating antidepressants)	Withdrawal only (4–8 biweekly 30 min sessions) vs. withdrawal (n = 23) + placebo biofeedback vs. withdrawal (8 weekly 45 min sessions) (n = 23) + CBT (8 weekly 45 min sessions) (n = 24)	Conversion of the dose of hypnotics in a number of “lowest recommended dosages” (LRD): gradual reduction to nightly dose at 1 LRD then gradual elimination of nightly dose	Drug-free patients at post-treatment: 67% CBT vs. 61% placebo feedback vs. 52% withdrawal only (ns) At follow-up (1 year): 50% vs. 35% vs. 43% (ns)

BZD: benzodiazepine; CBT: cognitive behavioral therapy; p.r.n: pro re nata; GAD: generalized anxiety disorders; NST: non-specific psychological treatment; LRD: lowest recommended dosage.

## Data Availability

No new data were created or analyzed in this study. Data sharing is not applicable to this article.

## Abbreviations

ACT	Acceptance and Commitment Therapy
BZD	Benzodiazepines and Z-drugs
CBT	Cognitive Behavioral Therapy
TAU	Treatment As Usual
SRD	Substance-related disorder
WL	Waiting List
